# Facile Synthesis and Life Cycle Assessment of Highly Active Magnetic Sorbent Composite Derived from Mixed Plastic and Biomass Waste for Water Remediation

**DOI:** 10.1021/acssuschemeng.2c04095

**Published:** 2022-09-07

**Authors:** Ahmed I. Osman, Ahmed M. Elgarahy, Neha Mehta, Ala’a H. Al-Muhtaseb, Ahmed S. Al-Fatesh, David W. Rooney

**Affiliations:** † School of Chemistry and Chemical Engineering, Queen’s University Belfast, Belfast BT9 5AG, Northern Ireland, United Kingdom; ‡ Environmental Science Department, Faculty of Science, Port Said University, Port Said 42526, Egypt; § Egyptian Propylene and Polypropylene Company (EPPC), Port-Said 42526, Egypt; ∥ Department of Petroleum and Chemical Engineering, College of Engineering, Sultan Qaboos University, Muscat 123, Oman; ⊥ Chemical Engineering Department, College of Engineering, King Saud University, Riyadh 11421, Saudi Arabia

**Keywords:** Plastic waste, Biomass waste, Adsorbent, Magnetic sorbent, Water remediation, Circular economy, Life cycle assessment

## Abstract

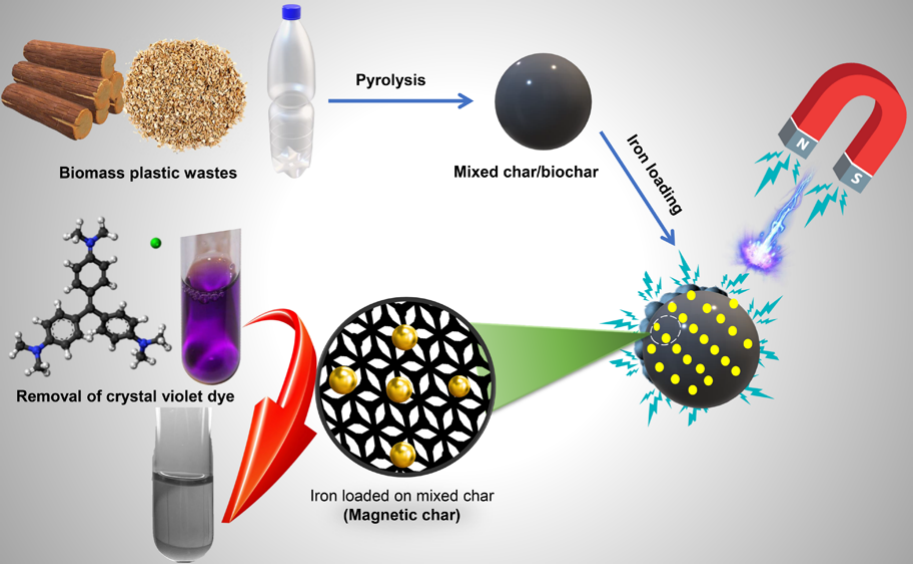

Plastic and biomass waste pose a serious environmental risk; thus, herein, we mixed biomass waste with plastic bottle waste (PET) to produce char composite materials for producing a magnetic char composite for better separation when used in water treatment applications. This study also calculated the life cycle environmental impacts of the preparation of adsorbent material for 11 different indicator categories. For 1 functional unit (1 kg of pomace leaves as feedstock), abiotic depletion of fossil fuels and global warming potential were quantified as 7.17 MJ and 0.63 kg CO_2_ equiv for production of magnetic char composite materials. The magnetic char composite material (MPBC) was then used to remove crystal violet dye from its aqueous solution under various operational parameters. The kinetics and isotherm statistical theories showed that the sorption of CV dye onto MPBC was governed by pseudo-second-order, and Langmuir models, respectively. The quantitative assessment of sorption capacity clarifies that the produced MPBC exhibited an admirable ability of 256.41 mg g^–1^. Meanwhile, the recyclability of 92.4% of MPBC was demonstrated after 5 adsorption/desorption cycles. Findings from this study will inspire more sustainable and cost-effective production of magnetic sorbents, including those derived from combined plastic and biomass waste streams.

## Abstract 2

Combining plastic and biomass waste streams in the synthesis of magnetic char composite material for dye removal in water treatment.

## Introduction

Plastics are used in various products and applications due to their cost-effectiveness, mechanical and chemical durability, flexibility, and versatility.^1−3^ The success of plastics is reflected in the global production rate escalating from 2 million tonnes (Mt) per year in 1950 to 350 Mt per year in 2015.^4^ This widespread production and use of plastics has also led to environmental issues associated with the disposal of plastic solid waste (PSW).^5,6^ For instance, approximately 8300 million metric tons of plastics have been produced until 2015, leading to generation of 6300 million metric tons of PSW between 1950 and 2015, of which 9% was recycled, 12% incinerated, and 79% disposed of in landfills or the natural environment.^7^ The global PSW generation rate was 1.3 billion tons per year in 2015, with an estimated increase to 2.2 billion tons per year by 2025, accompanied by an acceleration in the per capita waste production rate from 1.2 to 1.42 kg/person/day.^8^

The excessive use of plastic materials in numerous daily and industrial activities, along with the production of vast quantities of PSW, contributes to water, air, and land pollution.^9−13^ In addition, PSW tends to accumulate on beaches, diminishing their aesthetic and recreational value. The generated toxic debris (microplastics) from PSW can enter aquatic systems and accumulate through the food chain, posing risks to the environment, including plants, animals, and humans.^14^ The incineration of PSW releases chemicals (dioxides and phosgene) that are hazardous to the ecosystem. Dioxins released by plastic polymers are carcinogenic, persistent organic contaminants that pose grave risks to human health (e.g., cancer and neurological damage). Insignificant amounts of phthalates in children’s toys can cause serious health issues, including congenital diseases and malignant cancers.

Plastic bottles are mostly made of polythene terephthalate (PET), polycarbonate (PC), and high-density polythene (HDPE), with caps made of high density poly ethylene (HDPE), low density poly ethylene (LDPE), and polystyrene (PS). In the various bottling sectors, various polymeric materials, bottle forms, shapes, and colors are used.^15,16^ In 2019, about 58% of PET bottles were collected and sorted for recycling in the European Union (EU). In 2020, recycled PET (rPET) for bottle-to-bottle applications accounted for 32% of the European rPET market, with a recycled content of 14% on average.^17^

The limited supply of natural resources for the production of plastics and the harmful environmental impacts of plastic waste has led to the use of circular economy approaches. In this context, a circular economy refers to a framework that focuses on eliminating waste and pollution, circulating products and materials, and regenerating nature. Similarly, several thermal treatment strategies (i.e., gasification, pyrolysis, and incineration) have been developed for the conversion of plastic solid waste into energy and value-added products. Among them, pyrolysis is a well-known emergent, an as-designed process that operates by thermochemical conversion (heating) of as-used samples in an inert atmosphere to convert PSW into valuable products: gas (18–30 wt %), oil (22–49 wt %), and char (30–50 wt %), respectively.^4,18,19^

Deterioration of water quality and the rising demand for clean water have led to the development of many water treatment technologies.^20,21^ Organic dyes have been identified as one of the largest polluters of wastewater. Most dyes discharged into industrial effluent contain a complex of organic chemicals with high toxicity, such as aromatic compounds, amines, and traces of heavy metals, and are not biodegradable, even carcinogenic and mutagenic, posing major hazards to human health.^22−25^ Wastewater containing dyes is difficult to treat using traditional techniques because of the problems related to the removal of color.

Against this backdrop, Kumari et al. (2022) reported the preparation of activated carbon from plastic waste through carbonization and chemical activation processes. They demonstrated maximum sorption capacities of 16.28, 43.93, and 115.4 mg g^–1^ for P-ACs (waste polybags), C-ACs (cups), and B-ACs (bottles), respectively, toward thymol blue dye at basic pH of 9.0.^26^ Miandad et al. (2018) have inspected the sorptive characteristics of modified carbon derived from pyrolysis of polystyrene plastic waste (e.g., carbon–metal double layered oxides) for the adsorption of Congo red (CR). They revealed that the optimum equilibrium time was 180 min, whereas the acidic medium (pH ∼ 4.5) favored the maximum adsorption of CR up to 317.2 mg/g on the synthesized sorbent.^27^ Wei and Kamali (2021) have fabricated a mesoporous carbon nanostructure (MCN) embedded with Co_3_Fe_7_/CoFe_2_O_4_ magnetic nanoparticles derived from plastic waste via a clean and low-cost ball-milling/molten salt processing approach for efficient adsorption of cationic and anionic dyes. They illustrated that the as-employed ferromagnetic sorbent exhibited a good sorption capacity of 238.0 and 278.0 mg g^–1^ for methyl orange and methylene blue dyes, respectively.^28^ Li et al. (2021) demonstrated upcycling low-cost polyurethane plastic waste into activated carbon to remove the malachite green dye. They documented that the fabricated PUPW-AC sorbent has a maximum sorption capacity of tested dye on the PUPW-ACs was 1428 mg g^–1^.^29^

Therefore, herein, we mixed biomass waste with plastic waste to produce a magnetic char composite via pyrolysis for use in water treatment applications. To the authors’ best knowledge, this is the first comprehensive study on the production of magnetic char composite from plastic bottle waste and pomace biomass waste for crystal violet dye removal from water. Furthermore, this study also presents a life cycle assessment (LCA) of preparing a pomace leaves-based biochar composite adsorbent to appraise the environmental impacts of the preparation process. Life cycle assessment is a method for evaluating the environmental impacts of all stages of a commercial product, process, or service’s life cycle.^30^

## Materials and Methods

### Adsorbent Preparation

#### Preparation of Pomace Plant Supernatant

The pomace extract was employed as a reducing agent for synthesizing magnetite char composite nanoparticles. Pomace leaves were thoroughly *in situ*, rinsed twice with deionized water to remove dust and impurities, and then dried at 100 °C for 3 days. Then, the dried pomace leaves were crushed into powder. Next, 50 g of pomace powder was weighed and placed in a glass-boiling flask. Then, 100 mL of deionized water was added to the pomace powder. Then, the mixture of pomace powder and deionized water was heated on a shaking incubator for 2h at 75.0 °C. The powder extract was filtered under vacuum after reaching room temperature and stored in a refrigerator for further use in the green synthesis of magnetite char composite nanoparticles. Moreover, the residual pomace powder was mixed with ground plastic bottle waste pieces (feedstock to pyrolysis process).

#### Preparation of Plastic Waste-Biomass Char Composite (PW-BCC)

The mixture of residual pomace powder (60.0 g) and plastic bottle waste pieces (60.0 g) was sequentially followed by cleaning, drying, and grinding. The sifted plastic waste-biomass mixture (e.g., 1:1 wt %) powder was added to the quartz boat and pyrolyzed for 2.0 h at 550 °C in a tubular reactor under N_2_ flow (5 °C min^–1^ heating rate). After pyrolysis, the sample was allowed to cool to near room temperature; the biochar was collected, weighed, and stored in an airtight container.

#### Preparation of Magnetic Plastic Waste-Biomass Char Composite (MPBC)

Typically, the magnetic plastic waste-biomass char composite was prepared by mixing 25 g of plastic waste-biomass char composite with a 2:1 molar ratio of FeCl_3_·6H_2_O (e.g., 4.448 g) and FeSO_4_·7H_2_O (2.224 g) into a 250 mL round-bottomed flask containing 20 mL of pomace plant supernatant, and kept under stirring for 60 min. Afterward, the solution was heated at 80 °C while magnetically stirring for the later 30 min. Then, 30 mL of 1 M NaOH was added stepwise at 80 °C under constant stirring. The appearance of a black precipitate confirmed the successful synthesis of the magnetic plastic waste-biomass char composite (MPBC). Finally, the produced magnetic composite (solid) was magnetically collected from the solution using a neodymium magnet, rinsed with deionized water twice, and dried under vacuum for 24 h. For characterization purposes, pure magnetite was analyzed along with magnetic biomass char (MBC) and plastic biomass char (PBC).

### Adsorption Removal Experimentation

#### Standard Stock Solution of Crystal Violet Dye

A stock solution of 1000 mg L^–1^ of CV dye was prepared by dissolving dye powder in an appropriate water volume (total volume = 1.0 L). Standard working solutions of CV dye of various concentrations ranging from 10 to 1000 mg L^–1^ were obtained by diluting the stock solution. All adsorption experiments were repeated three times to ensure reproducibility and were reproducible within 2% error at most.

#### Experimental Setup

In a 150 mL Erlenmeyer flasks containing 20.0 mL aqueous solution were carried out batch mode sorption experiments for CV dye on the MPBC surface. During the sorption process, the influence of experimental variables such as initial medium pH (e.g., 2.3 to 10.5), sorbent mass (e.g., 0.01 to 0.1 g), initial dye concentration (e.g., 10 to 1000 mg L^–1^), reaction time (e.g., 3.0 to 180.0 min), environmental surrounding temperature (e.g., 298.0 to 320.0 K), and the presence of competitive ions was systematically observed. All sorption processes were conducted in a shaking incubator (LSI-3016R, LabTech S.r.l., Sorisole (BG), Italy) with 150.0 rpm at room temperature 25 ± 1°C. At the end of different sorption trials, the as-employed sorbent was magnetically separated using an external neodymium magnet. The residual dye concentrations after the sorption process were analyzed using a spectrophotometer (Palintest 7100 spectrophotometer, Palintest Ltd., Gasteshead, UK) at λ = 584.0 nm wavelength.^31^ The mass balance sorption capacity and removal efficiency (RE %) of CV dye was measured by using eq 1 and 2.
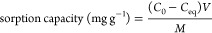
1
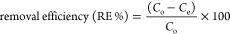
2where *q*_e_ is the dye sorbed amount by the MPBC sorbent weight, *C*_0_ = initial dye concentration and *C*_eq_ = final dye concentration of a tested aqueous solution in mg L^–1^, *V* = volume of solution in Liter, and *M* = sorbent mass in g.

All sorption tests were performed three times, and the averages were recorded. The limit of experimental errors on triplicates was systematically below 2%.

The reusability of the MPBC sorbent was checked by employing subsequent processing cycles. Based on the cationic nature of the CV dye, HCl was selected as a desorption medium to desorb CV dye molecules from the exterior surface of the MPBC sorbent. At the end of the sorption process, the dyes laden-sorbents were simply retrieved from the dye solution, washed with deionized water, and suspended in 0.5 M HCl to desorb the sorbed CV dye molecules, respectively. The admixture was agitated for 60.0 min, and the regenerated MPBC sorbent was separated, rinsed with deionized water, dried, and used for another sorption cycle. Following the same pattern, the sorption, desorption and resorption were regularly repeated for 5 consecutive cycles. The desorption efficiency (DES %) of MPBC sorbent can be estimated using eq 3.
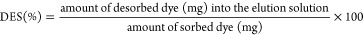
3

The cooperative use of the MPBC sorbent to remove the CV dye from real textile dyeing effluents was conducted, with wastewater collected from the discharge stream of a local dyeing plant at industrial Zone, Port-Said, Egypt, as the background. The sample was sieved through a 130.0 μm to remove any suspended materials. The collected wastewater sample was spiked with different concentrations of CV dyes (e.g., 5.0 to 20.0 mg L^–1^). Then, the spiked wastewater liquor (0.02 L) was treated with 1.5 g L^–1^ of the MPBC sorbent at room temperature of 25 °C ± 1, contact time of 150.0 min, and stirring speed of 150.0 rpm. The specimen was filtered, and the supernatant was analyzed to deduct the removal efficacy of the MPBC sorbent for the CV dye from wastewater.

## Characterization Techniques

The composite char materials were characterized using X-ray powder diffraction (XRD), X-ray photoelectron spectroscopy (XPS), transmission electron microscopy energy-dispersive X-ray spectroscopy (TEM-EDX), and ζ-potential, which are provided in detail in the Supporting Information.

## Results and Discussion

### Characterization Results

**Figure 1:**
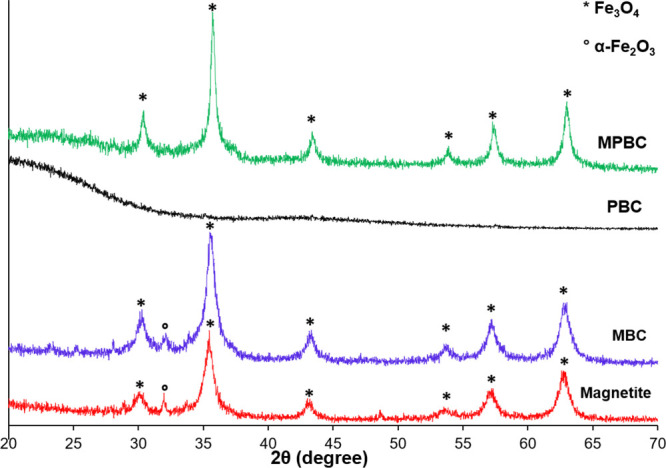
XRD diffraction patterns of char composite samples of magnetic and nonmagnetic composite chars.

The XRD patterns of the synthesized magnetic and nonmagnetic composites char are shown in Figure 1. The magnetite samples showed diffraction peaks attributed to Fe_3_O_4_ ((JCPDS No. 03-0863) at 2θ of 30.3° (220), 35.4° (311), 43.4° (400), 53.5° (422), 56.9° (511), and 62.6° (440),^32,33^ with a small diffraction peak at 2θ of 32° corresponding to α-Fe_2_O_3_. The nonmagnetic char of biomass and plastic only (PBC) did not show any diffraction lines related to Fe_3_O_4_, while the magnetic char composites of MBC and MPBC samples showed only diffraction lines for Fe_3_O_4_.

The surface charge is essential to the adsorption mechanism; consequently, ζ-potential was utilized in this study. The tendency of particles with the same electrical charge to repel each other is proportional to the ζ-potential. A higher negative ζ-potential indicates higher repulsion between nanoparticles and a lower tendency to agglomerate in the nanofluid.^34^ The magnitude of the ζ-potential is very important in determining the stability of the nanoparticle systems. In general, particles having the ζ-potential values higher than +30 mV or lower than −30 mV are considered as stable suspensions.^35,36^ Due to the mutual repulsion of magnetic, MBC or MPBC surface charges, char magnetic composite samples exhibited a high average value of ζ-potential, with MPBC and MBC exhibiting a greater negative charge (∼ −35 mV) than the magnetite samples (∼ −28 mV).

**Figure 2:**
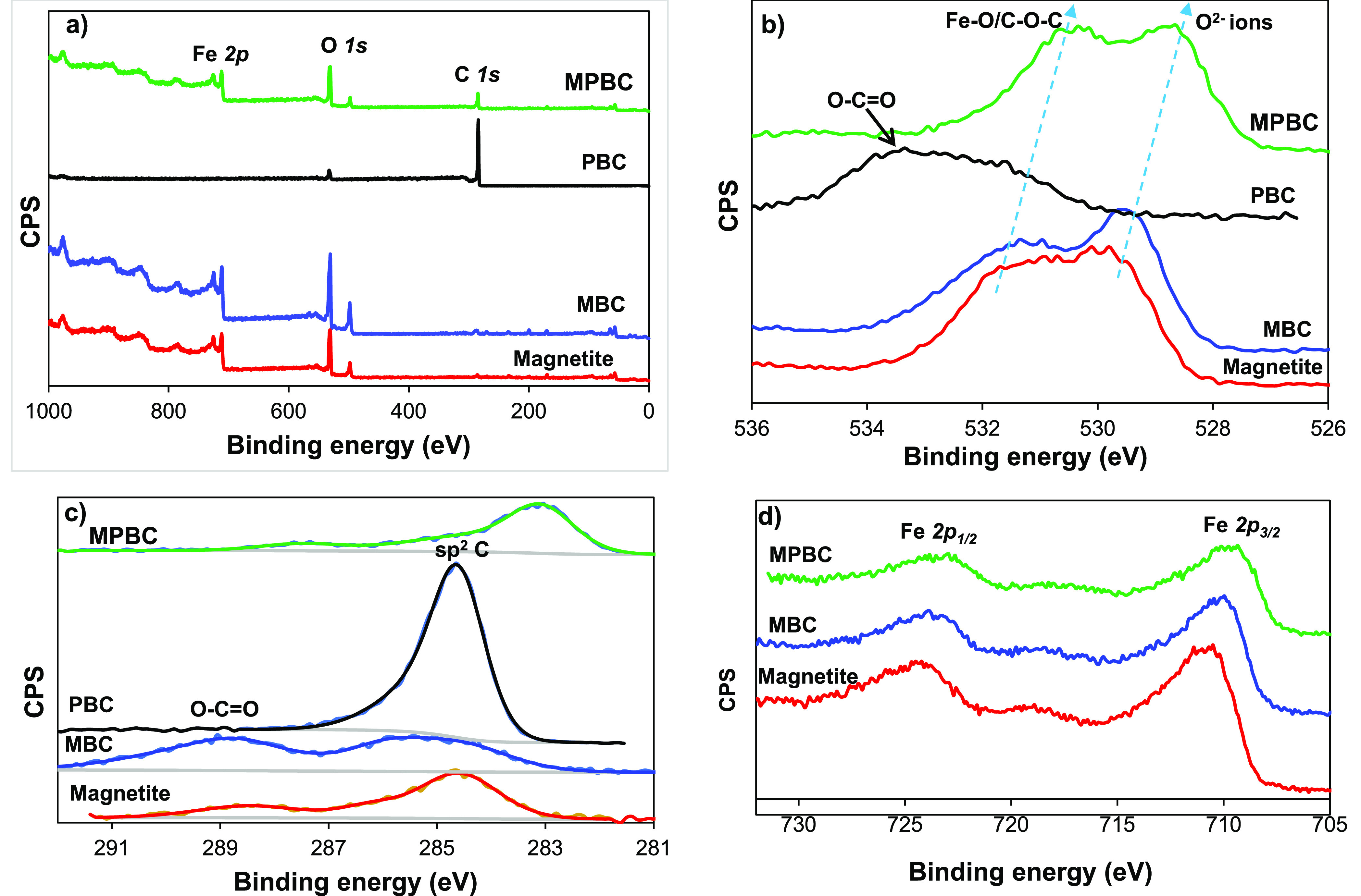
XPS spectra of magnetic and nonmagnetic composite chars. XPS survey (a) and XPS spectra in the (b) O *1s*, (c) C *1s*, and (d) Fe *2p* regions.

The XPS spectra of magnetic and nonmagnetic composite chars were performed to investigate the surface oxidation of the char composites, as shown in Figure 2. The XPS survey shows XPS spectra for O *1s*, C *1s*, and Fe *2p*. The magnetite samples showed two XPS spectra for O *1s* at 529.3 eV (O^2–^) (which shifted to a lower binding energy at 528.7 eV for MPBC) and a peak at 531.2 eV (Fe–O/C–O–C) (which also shifted to lower binding energy at 530.4 eV for MPBC). This indicates that the iron oxide (Fe_3_O_4_) particles were embedded in the char composite surface. The nonmagnetic char composite (PBC) showed only a peak centered at 533.6 eV, which is attributed to the O=C—O bonding in the char composite structure.^37^ The C *1s* spectra of the magnetite char composites mainly two peaks at 284.6 eV for sp^2^ C (shifted to a lower binding energy of 283.1 eV for MPBC) and 288.7 eV for O—C=O. Finally, the Fe *2p* spectra showed two peaks at 710.4, and 724.2 eV, which are attributed to the Fe *2p*_*3/2*_ and Fe *2p*_*1/2*_ states, respectively, implying the presence of Fe_3_O_4_ within the magnetic char composites.

**Figure 3:**
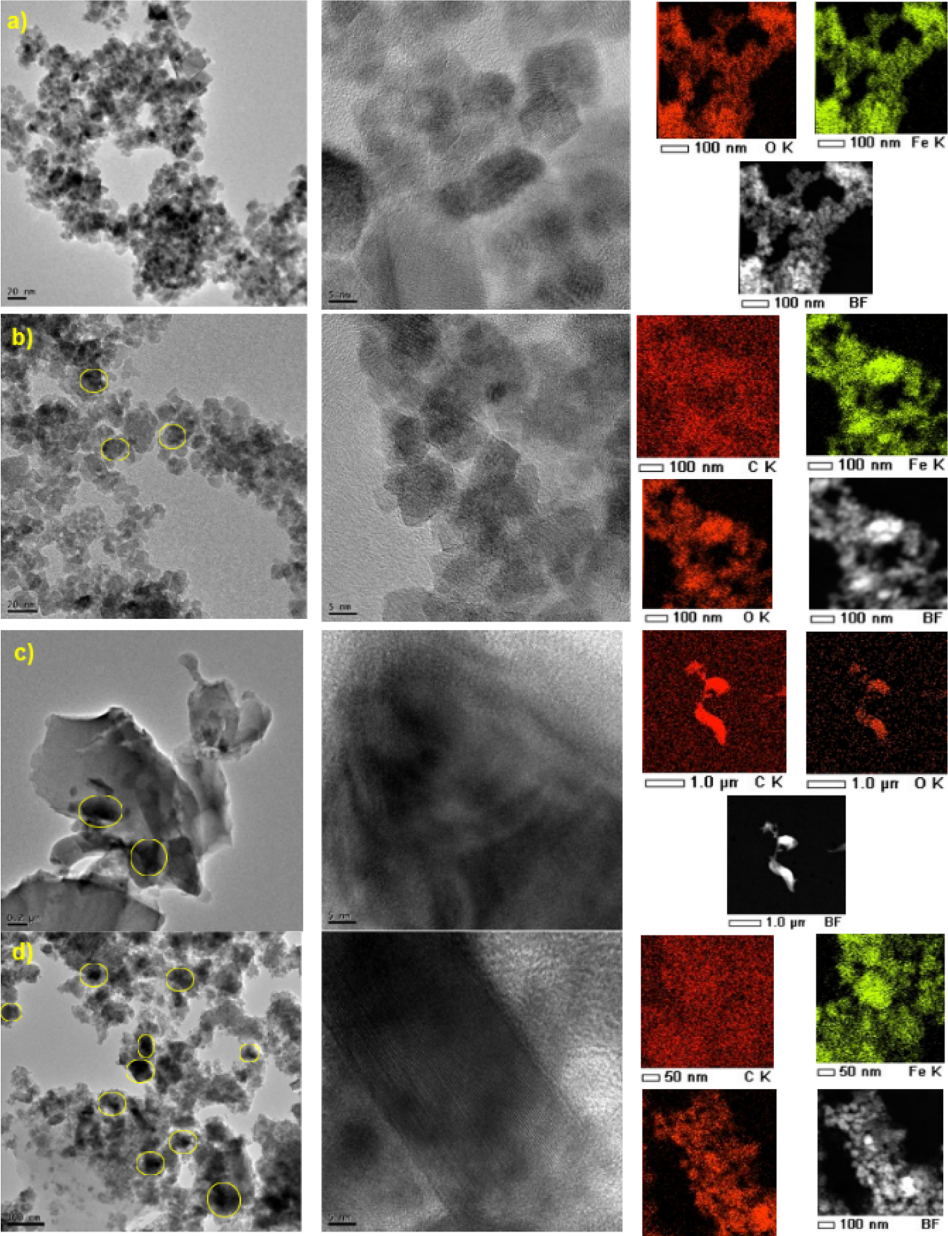
TEM images and elemental mapping of (a) pure magnetite, (b) magnetite and biomass (MBC), (c) plastic biomass char composite (PBC), (d) magnetite mixed plastic biomass char composite (MPBC).

Figure 3 depicts the TEM photographs morphology of the char composites along with the EDX elemental mapping. Magnetite sample exhibited spherical and square particle shapes; however, after incorporation into biomass char, particle shapes are predominantly spherical with good dispersion of iron nanoparticles in the char composite structure shown in the elemental mapping (Figure 3b). In contrast, the PBC exhibited a lumpy structure. Figure 3d demonstrates that the MPBC had smaller particle sizes of <5 nm with a good iron particle size distribution.

### Life Cycle Assessment Results

**Figure 4:**
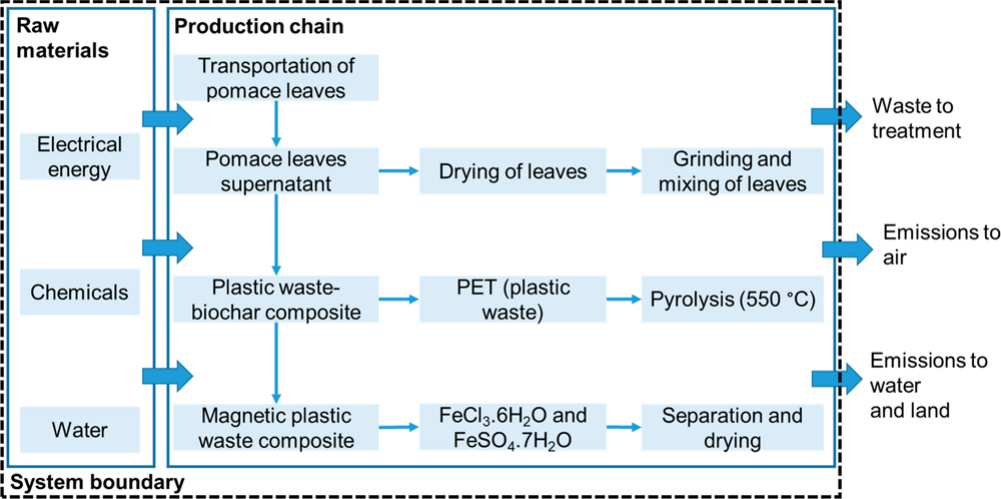
System boundary considered for producing magnetic plastic waste-biomass char composite material.

In this study, the goal of using LCA was to calculate the environmental impacts of the production of magnetic char composite adsorbent material following the procedures and guidelines defined in ISO 14040:2006 and ISO14044:2006 standards.^38,39^ SimaPro v9 software and the Ecoinvent database were used to conduct the LCA for the cradle-to-gate attributional approach (Figure 4) and excluded environmental impacts due to infrastructure processes. The functional unit was 1 kg of pomace leaves used to prepare the adsorbent material. The midpoint indicator impact assessment was carried out according to the CML-IA Baseline method.

This study investigates using pomace leaves residues and plastic waste to prepare magnetic char composite adsorbent material. The goal of using LCA was to evaluate the environmental impacts of the adsorbent material production chain.

#### Inventory Analysis

The system boundary did not include the production of plastics (PET) and pomace leaves, as both were considered to be abundantly available waste-based feedstocks. For 1 kg of pomace leaves, 649 g of plastic waste (PET) was utilized for the entire process. The total transportation distance for pomace leaves and plastic waste was considered to be 100 km for each.

For supernatant preparation, pomace leaves residues, electrical energy, and deionized water were required. About 188 g of pomace leaves was utilized for supernatant preparation in a quantity of 301 mL, as shown in Table 1. Deionized water required for cleaning pomace leaves was considered to be in the same quantity as pomace leaves, that is, 188 g for 188 g of pomace leaves (considering the density of deionized water as 1000 kg/m^3^). It was assumed that the pomace leaves had an initial moisture content of 20% after cleaning. The supernatant preparation process (Adsorbent Preparation Section) used an oven for drying leaves; however, it is very likely that for large-scale production, a commercial leaves dryer would be in operation. The electrical energy requirement for drying leaves was parametrized according to the specific energy mentioned in Ye et al.^40^ as 0.8 MJ kg^–1^. Furthermore, the electrical energy requirement for grinding dried leaves was modeled in line with Lomovskiy et al.^41^ Deionized water needed for preparing the supernatant was quantified as 301 g (Adsorbent Preparation Section). The electrical energy for raising and maintaining the temperature of the mixture from 20 and 80 °C was estimated as 0.6 MJ.^42^ The energy and material required for vacuum filtration were assumed to be negligible.

**Table 1:** Inventory Data for Conducting Life Cycle Assessment to Prepare Magnetic Adsorbent Material Using 1 kg of Pomace Leaves

Material/Process	Unit	Input	Output	Reference
Transportation^a^				
Transportation of pomace leaves	t·km		0.10	Calculation (1-tonne·km distance)
Transportation of plastic waste	t·km		0.06	
Pomace leaves supernatant preparation				
Pomace leaves	g	188.3		
Deionized water for cleaning leaves^b^	g	188.3		
Electrical energy for drying leaves^c^	MJ	0.15		(40)
Electrical energy for grinding leaves	10^6^ MJ	9.0		(41)
Deionized water for supernatant	g	301.3		
Electrical energy for heating at 75 °C	MJ	0.6		(42)
Supernatant	g		301.3	
Pyrolysis				
Pomace leaves	g	811.8		
Deionized water for cleaning leaves	g	811.8		
Electrical energy for drying and grinding leaves	MJ	0.65		(40, 41)
Plastic waste (PET)	g	649.4		
Electrical energy for the pyrolysis	MJ	0.001		(43, 44)
Composite material	g		376.6	
Magnetic plastic waste-biomass char composite				
Composite material	g	376.6		
FeCl_3_·6H_2_O^d^	g	67		
FeSO_4_·7H_2_O^e^	g	33.5		
Supernatant	g	301.3		
Electrical energy for heating at 80 °C	MJ	3.04		(42)
Deionized water for the solution	g	452		
NaOH^f^	g	0.22		
Magnetic composite material (after 10% losses)	g		428.7	
Deionized water for cleaning	g	428.7		
Energy for drying in the oven	MJ	0.05		(45)

a Transport, freight, lorry 3.5–7.5 t, euro6/market for transport, freight, lorry 3.5–7.5 t, EURO6 | APOS, U.

b Deionized water, reverse osmosis, production mix, at the plant, from groundwater RER S.

c Electricity grid mix 1–60 kV, AC, consumption mix, at consumer, 1–kV EU-27 S.

d Pig iron^46^| market for | APOS, S; chlorine gas, production mix/RER Mass; deionized water, reverse osmosis, production mix, at the plant, from groundwater RER S.

e Pig iron^46^| market for | APOS, S; sulfuric acid | market for sulfuric acid | APOS, U; deionized water, reverse osmosis, production mix, at the plant, from groundwater RER S.

f Sodium hydroxide, chlor-alkali production mix, at plant/RER.

To prepare the plastic waste-biomass char composite, pyrolysis at 550 °C was carried out. About 649 g of pomace leaves powder, and 649 g of plastic waste were pyrolyzed to produce 377 g of the plastic waste-biomass char composite (yield of char composite was taken as 29%). The need for heat and power of the pyrolysis unit is about 12% of the energy content of input, which would be equivalent to 21.28 kJ kg^–1^ in the present scenario.^43,44^

Furthermore, it was quantified that for 377 g of plastic composite and 301 g of supernatant, about 67 g of FeCl_3_·6H_2_O and 33.5 g of FeSO_4_·7H_2_O were required. It should be noted that since the Ecoinvent database did not present the environmental impacts of these chemicals, it was assumed that these chemicals would be prepared by using pig iron and chlorine gas and pig iron and sulfuric acid. The energy requirement for heating the solution to prepare the magnetic composite was estimated as 3.03 MJ.^42^ About 0.06 g of NaOH and 452 mL of deionized water were also added to the solution while stirring. The energy required for stirring was considered to be negligible. After the process, it was assumed that there would be 10% losses in recovering the magnetic adsorbent material. Moreover, 1 kg of deionized water was conservatively estimated to be needed to clean the magnetic adsorbent material. Electrical energy was calculated for drying the magnetic adsorbent material according to equipment energy requirements.^45^

#### Environmental Impact Assessment and Interpretation

Midpoint indicator assessment was carried out to understand the environmental impacts of the production chain using the CML-IA baseline method. All the environmental impacts were measured for 1 functional unit, that is, utilization of 1 kg of pomace leaves as the feedstock.

#### Abiotic Depletion of Resources and Fossil Fuels

Abiotic depletion is the decrease of availability of the total reserve of functions of resources such as fossil fuels, minerals, clay, and peat. Abiotic depletion is measured in kilograms of antimony (Sb) equivalents (equiv). Total abiotic depletion of resources was observed as 1.7 × 10^–7^ kg Sb equiv, with the highest impacts observed for magnetic plastic waste-biomass char composite 1.5 × 10^–7^ kg Sb equiv (about 90% of the total). This is because of the environmental impacts of chemicals (iron ore based).

Abiotic depletion of fossil fuels represents the overextraction of fossil fuels. Abiotic depletion of fossil fuels was also highest for the magnetic composite material preparation (5.1 MJ) due to the use of electrical energy for preparing the solution and drying in the oven and raw materials such as chemicals. Abiotic depletion for transportation of pomace leaves and plastic waste was 1 MJ.

#### Global Warming Potential

Global warming potential in this study was accounted for a 100-year-horizon due to the emissions of greenhouse gases in the production chain. The total global warming was noted as 0.63 kg CO_2_ equiv. The highest global warming potential (0.46 kg CO_2_ equiv) was also observed for magnetic plastic waste-biomass char preparation. This can be explained as this corresponds with the use and ultimate depletion of fossil fuels. Also, this was the only process that used chemicals such as FeCl_3_·6H_2_O and FeSO_4_·7H_2_O.

#### Air Pollution (Ozone Layer Depletion and Photochemical Oxidation)

Ozone layer depletion in the atmosphere is caused due to the release of foaming and cleaning agents and is measured in kg CFC-11 equiv. It was observed as 5.2 × 10^–8^ kg CFC-11 equiv for the entire process.

Photochemical oxidation accounts for the creation of the ozone in the presence of sunlight, nitrogen oxides, and volatile organic compounds. It was observed as 14 × 10^–5^ kg C_2_H_4_ equiv as a total for all the phases from transportation to adsorbent material preparation.

#### Toxicity-Related Impacts (Human Toxicity, Freshwater Aquatic Ecotoxicity, Marine Aquatic Ecotoxicity)

Toxicity-related impacts reflect the potential harm of a unit of chemical released into the environment. All four toxicity potentials were highest for the adsorbent material preparation stage. This is because of the use of pig iron and other chemicals in the process. Toxicity due to metals production processes are governed by the energy intensity and fuel mix, disposal of sulfidic tailings or emissions of toxic or acidifying pollutants to air, soil, and water.^47^

#### Water and Soil Pollution (Eutrophication and Acidification)

Eutrophication occurs due to an overload of nutrients in soil and water. Eutrophication potential for supernatant preparation and adsorbent material preparation was observed as 2.4 × 10^–5^ and 13.7 × 10^–5^ kg PO_4_^3–^ equiv, respectively.

**Figure 5:**
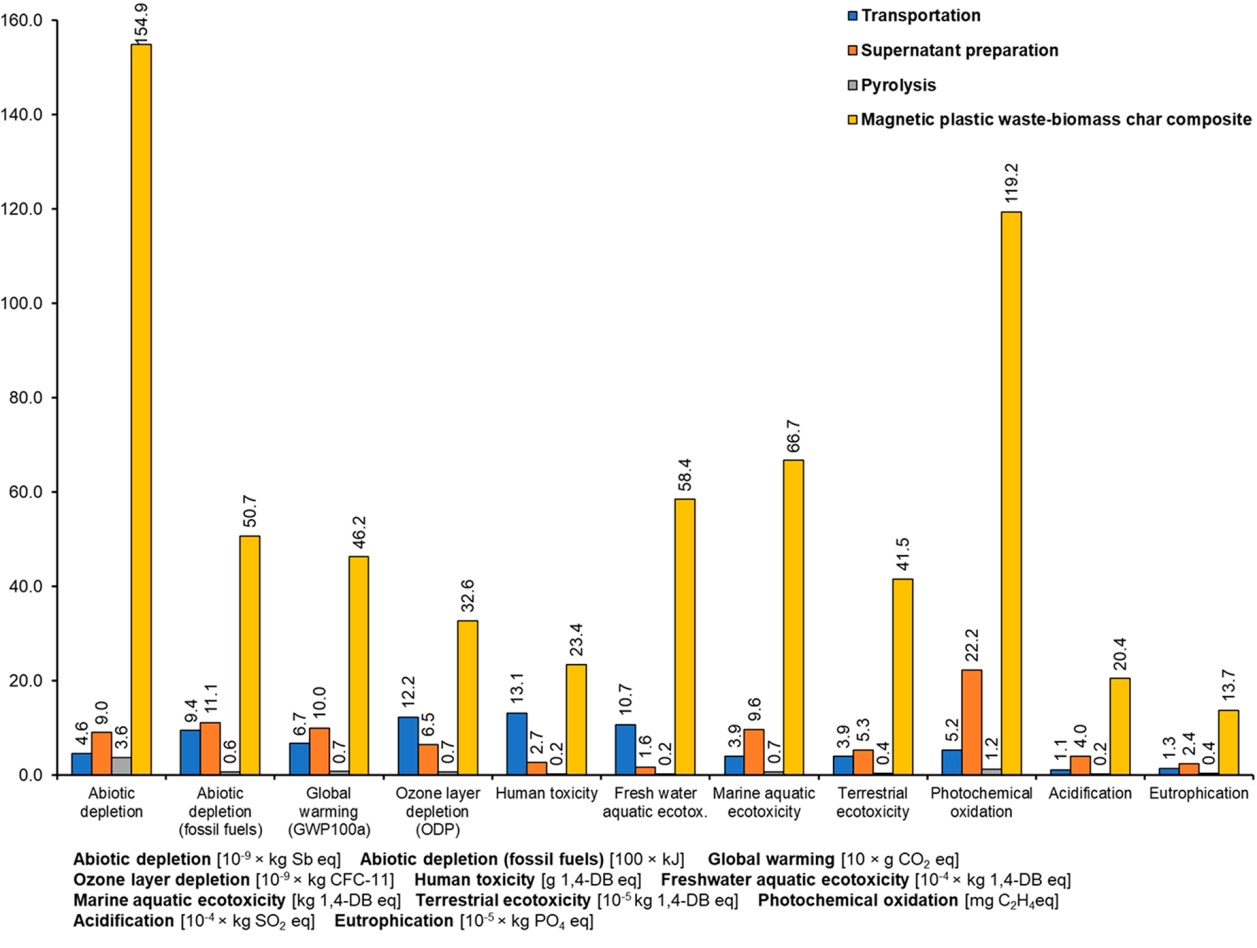
Comparison of environmental impacts for four different magnetic adsorbent material preparation stages.

Acidification potential measures the emissions, such as sulfur dioxide and nitrogen oxides from manufacturing processes. Here, 0.002 kg SO_2_ equiv was the acidification potential for the entire process (Figure 5).

### Adsorption Results

#### Crystal Violet Sorption in Batch Mode

This study examines the sorption of the CV dye from an aqueous solution onto the MPBC sorbent in batch mode. Throughout the entire examination, the following operational parameter effects were investigated.

#### Influence of Initial pH

**Figure 6:**
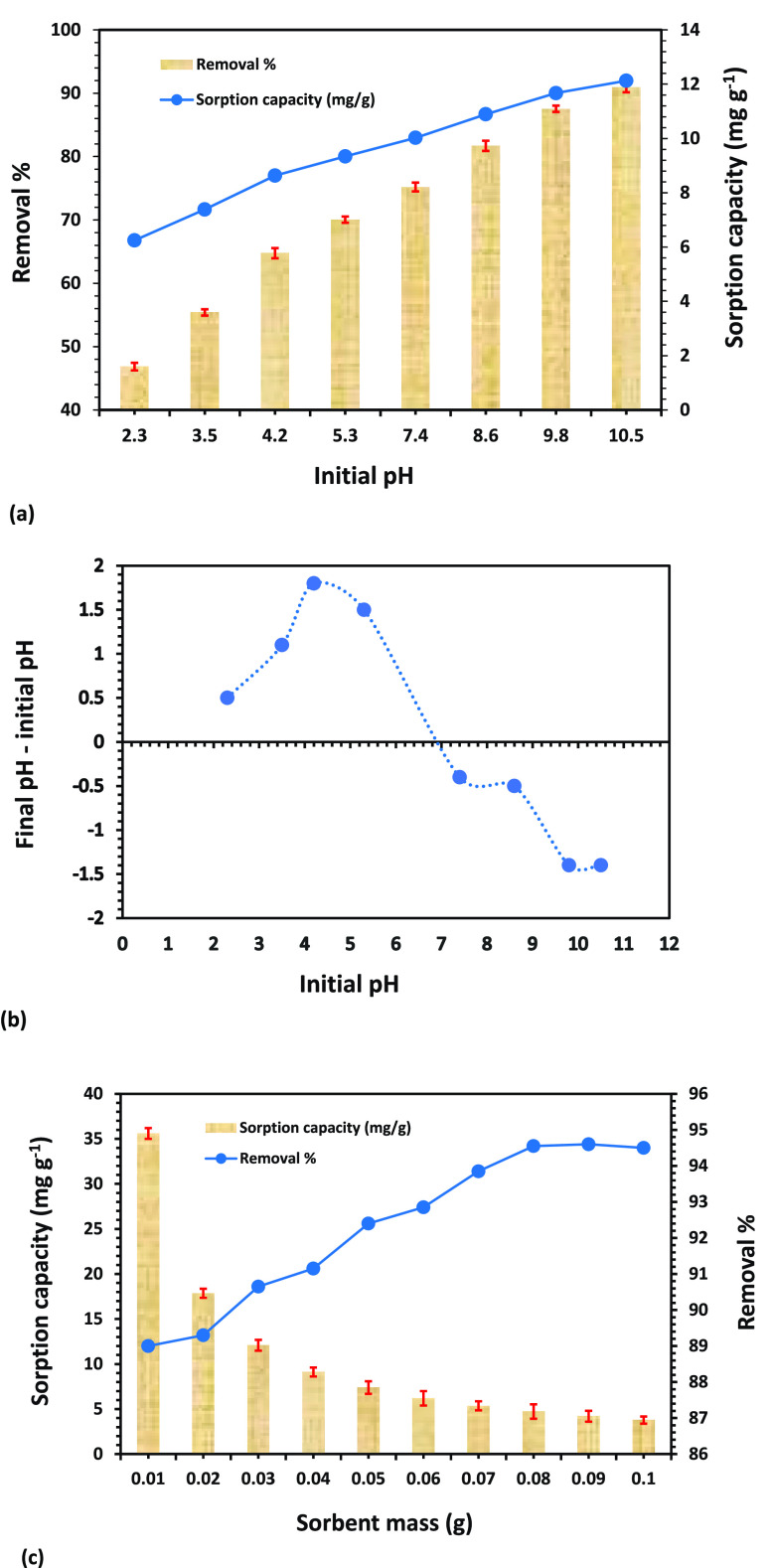
CV dye removal tests. Panels a and b are initial pH versus the loading capacity and final pH values, and panel c is sorbent dosage versus the loading capacity of the char composite.

In essence, the efficacy of a sorbent to sorb target water pollutants (e.g., ions/molecules) greatly depends on the acidity/basicity of the working solution. Concerning the ionic speciation forms of target pollutants, the pH of the solution determines the surface charge of the sorbent, which is essential for the sorption process.^24,48,49^ The experiment for CV dye sorption on the MPBC sorbent was conducted in the range of 2.3 to 10.5 in order to optimize the pH. A sorbent dose of 0.03 g was added to 20 mL of solution and agitated for 180.0 min at 25 ± 1 °C at room temperature. The sorption capacity and RE % of the CV dye increased from 6.2 (RE % = 46.8%) to 12.1 mg g^–1^ (RE % = 90.9%), with an increase in the initial solution pH from 2.3 to 10.5 as demonstrated in Figure 6a. In the acidic environment, the low RE % of the MPBC sorbent is attributed to the electrostatic repulsive forces between the protonated oxygen/nitrogen-containing functional constituents (positively charged surface) and the cationic fractions of the CV dye in the aqueous solution. Contrarily, the utmost sorption capacity and RE % of the MPBC sorbent toward the CV dye in the alkaline medium is because of attractive electrostatic forces between adversely charged species of positively charged CV dye and deprotonated sorbent surface (negatively charged). A similar phenomenon was reported during the sorption of the CV dye onto eco-friendly palm petiole-derived biochar.^50^

Given that the CV has two acid dissociation constants (p*K*_a_) values of p*K*_a__1_ 5.31 and p*K*_a__2_ 8.64, it is anticipated that the removal of CV at greater pH values will be more successful.^51^ As a result, the improved CV removal onto the completely ionized sorptive centers at basic pH is consistent with its dissociation constants. Moreover, the cationic nature of CV dye promoted its removal at higher pH values.^52^ Furthermore, the MPBC sorbent has no net charge (point of zero charge) of 7.0, as illustrated in Figure 6b. The measured pH_zpc_ value demonstrates the electrokinetic performance of the sorbent, which majorly depends on the type of sorbent material as well as the experimental synthesis conditions.^53^ When solution pH decreased below pH_zpc_, the surface charge was positively charged, and cationic CV dye and sorbent surface repel one another, which declines the efficiency of CV dye elimination. While, beyond the pH_zpc_, the surface charge was negatively charged, which admirably sorbed the cationic CV dye molecules.^54^ These findings displayed that a basic pH could boost the CV removal process. However, the as-employed MPBC sorbent exhibited a high RE % toward CV dye even at a solution pH value of 2.3, which proposed that sorption of CV dye onto MPBC sorbent may be dominated by other impactful mechanisms of pore-filling, H-bonding, and π-π stacking.^55−57^

#### Influence of Sorbent Concentration

To determine the optimal sorbent concentration for removing the CV dye from an aqueous solution, sorption was performed at a constant temperature with varying the sorbent concentrations (e.g., 0.01 to 0.1 g). As shown in Figure 6c, under constant pH, temperature, and sorbate mass, the RE % of CV uptake increases from 89.0 to 94.6% at a certain point and then remains constant as the sorbent dosage is increased. Initially, the sorbent exhibited fewer active sites to capture the dye molecules at low concentrations; however, as the sorbent doses increased, so did the RE %, as well as the development of more active sites on the MPBC sorbent surface and an increase in its surface area for the sorption of the CV dye.^58,59^ Up to 4.0 g L^–1^ of sorbent concentration, the RE % increased; thereafter, it remained constant as the sorbent dose increased; this phenomenon may be attributed to surface agglomeration.^60^ Therefore, 4.0 g L^–1^ of the sorbent is shown to have the optimized sorption for 20.0 mg L^–1^ of CV dye sorbate. When the majority of the sorbate molecules are present at the active sites, there is no chance of sorption, regardless of how much sorbent is employed.

#### Influence of Primary CV Concentration (Isotherms Assay)

**Figure 7:**
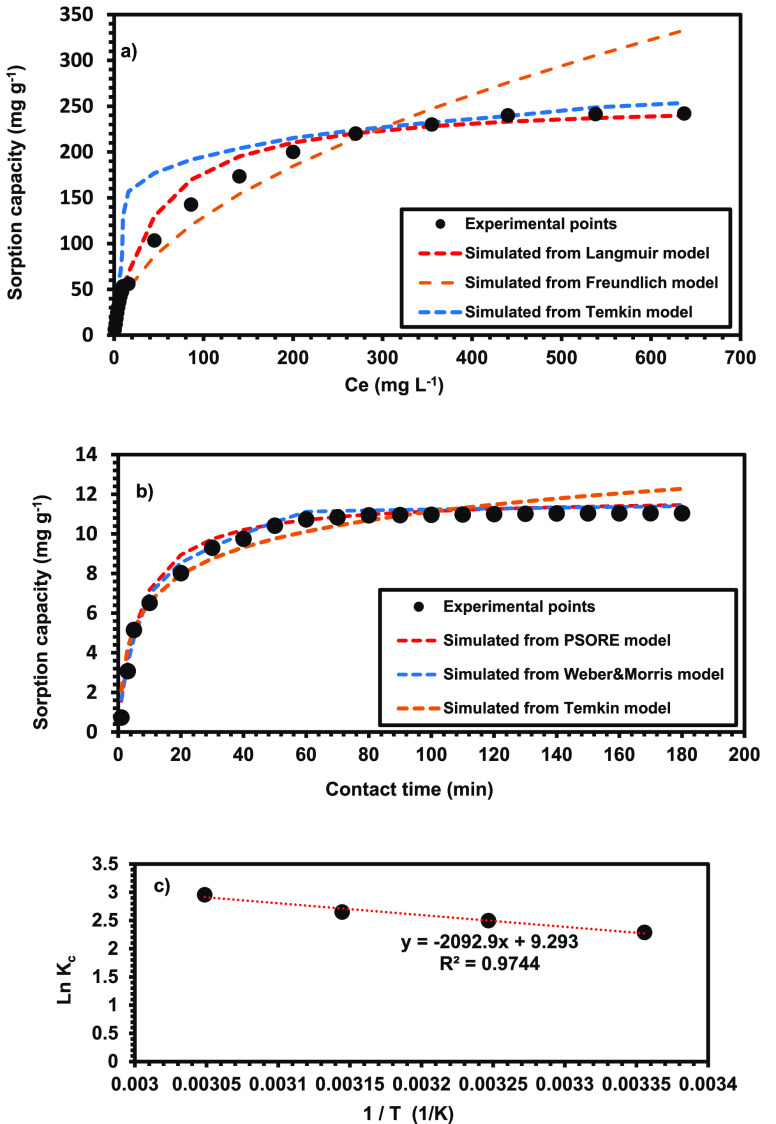
Experimental points of composite char in CV removal versus simulated models. It shows (a) *C*_e_ (mg L^–1^) and (b) contact time versus the sorption capacity in mg g^–1^. Panel c shows the inverse of temperature (1/*T*) against ln *K*_c_.

The influence of initial CV dye concentration on the removal process using the MPBC sorbent was inspected from 10.0 to 1000.0 mg L^–1^ under fixed operational parameters of pH = 10.5, sorbent dosage = 0.03 g, and interaction time = 180.0 min. The sorption capacity of the MPBC sorbent toward the CV dye gradually increased with an increase in the initial CV concentration and finally attained saturation at 1000.0 mg L^–1^ (Figure 7a). This could be attributed to the availability of the number of accessible vacant sorptive sites because of the porous structure of the MPBC sorbent surface. As the CV concentration increased, the number of effective collisions between CV molecules and MPBC sorbent carried out, and thence more and more dye molecules were captured within the binding sites of MPBC (concentration gradient phenomenon), which consequently acted as a main driving force, improved the sorption capacity of MPBC sorbent in single-phase medium, and then slowed down due to no more sites available for sorption process.^61,62^

The equilibrium findings derived from the sorption of CV dye (liquid) onto MPBC sorbent (solid) were simulated using three commonly standard isotherm models viz, Langmuir,^63^ Freundlich,^64^ and Temkin^65^ models. The isotherms are used to calculate the quantity of sorbate adsorbed on the sorbent surface from a solution. According to Langmuir’s assumption, sorption occurs at particular locations on the sorbent surface, and the adsorption energy is spread uniformly over the sorbent surface. The linear form of the model is represented in eq 4:
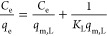
4where *q*_e_ (mg g^–1^) is the quantity of CV loaded on the MPBC sorbent at equilibrium, *q*_max_ (mg g^–1^) is the maximum Langmuir sorption capacity, and *K*_L_ (L mg^–1^) is the constant associated with the binding site affinity. The experimental data of *q*_max_, *K*_L_, and *R*^2^ were determined using a linear design between *C*_e_/*q*_e_ vs *C*_e_. The derived isotherm parameters and correlation coefficient (*R*^2^) are presented in Table 2. Furthermore, the dimensionless constant called equilibrium parameter *R*_L_, written as an equation, can clarify the key properties of the Langmuir isotherm:
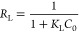
5where *R*_L_ denotes the nature of the sorption process, whether the sorption isotherm is unfavorable (*R*_L_ > 1) or favorable (0 < *R*_L_ < 1).

**Table 2:** Isothermal Modeling Parameters of CV Dye Sorption onto MPBC Sorbent

Isothermal models		Results
Langmuir	*k*_L_ (L mg^–1^)	0.02
	*q*_m_ (mg g^–1^)	256.41
	*R*^2^	0.99
Freundlich	*n*	1.96
	*k*_f_ (mg g^–1^) (L mg^–1^)^1/*n*^	12.47
	*R*^2^	0.96
Temkin	*A* (L g^–1^)	0.52
	*B* (kJ mol^–1^)	60.19
	*R*^2^	0.96

The multilayer sorption of CV dye molecules on the heterogeneous surface of the MPBC sorbent was postulated by the Freundlich isothermal model. The linear form of the Freundlich model is displayed as follows
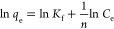
6where *K*_f_ (mg g^–1^) (L mg^–1^)^1/*n*^ is the Freundlich isotherm constant related to the sorbent sorption capacity, where *n* is the heterogeneity factor. A linear plot between ln *q*_e_ vs ln *C*_e_ was used to derive the values of *K*_f_, *n*, and *R*^2^. For optimal sorption, the value of *n* should be between 1 and 10.

The Temkin isotherm model assumes that the heat of sorption of all sorbate sorbed molecules/ions in the layer falls linearly with sorbent surface coverage because of contact. A linear form of the Temkin model is provided in eq 7:
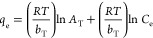
7where *R* is the universal gas constant (8.314 J mol^–1^ K), *T* (K) represents the absolute temperature, *b*_T_ (kJ mol^–1^) is the Temkin isotherm constant related to heat sorption, and *A*_T_ (L g^–1^) represents the equilibrium binding constant. The values of *b*_T_, *A*_T_, and *R*^2^ were obtained by plotting the graph between *q*_e_ vs In *C*_e_.

Among the three sorption isotherm models, the Langmuir isotherm model was shown to be the best fit in experimental data of CV sorption onto the MPBC sorbent. The Langmuir model’s *R*^2^ is 0.99, which is higher than the Freundlich (e.g., 0.962) and Temkin (e.g., 0.960), as shown in Table 2. Similar results were reported for magnetic sorbent-pollutant systems in the literature.^66−68^ In addition, the calculated *R*_L_ values ranged from 0 to 1 (e.g., from 0.04 to 0.81), confirming that the CV dye sorption process onto the MPBC sorbent is favorable.

#### Influence of Contact Period (Kinetics Assay)

The impact of reaction time on the CV dye decontamination using the MPBC sorbent was evaluated at different time intervals (e.g., 3.0–180.0 min). With an increase in contact time, the RE % sharply increased from 5.5 to 90.7%, as shown in Figure 7b. This may be due to increased electrostatic interaction between the CV dye molecules and the MPBC surface. Initially, a high removal rate of CV dye using MPBC sorbent was noticed in the first few minutes, which is attributed to the availability of more vacant sorptive sites. After that, the removal rate increased sluggishly with increasing contact time until it stabilized after approximately 60.0 min. This is demonstrated by a decrease in free binding sites as the surface of the sorbent became saturated with dye molecules, and no additional sorption was detected after the equilibrium stage.^69^

Kinetic fitting is employed for acquiring a comprehensive understanding of CV sorption onto the MPBC sorbent, in addition to defining the rate-controlling phase that is majorly responsible for CV dye sorption. The sorption kinetics parameters were examined using four kinetic models; pseudo-first-order (PFORE),^70^ pseudo-second-order (PSORE),^71^ intraparticle diffusion model (IPD),^72^ and Elovich^73^ models, respectively, as demonstrated in eqs 8–11 (linear forms).
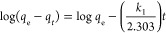
8
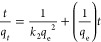
9
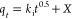
10
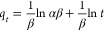
11where *q_t_* (mg g^–1^) is the amount of dye sorbed at time (*t*), *q*_e_ (mg g^–1^) is the equilibrium sorption, *K*_1_ (min^–1^) is a pseudo-first-order rate constant of sorption, *K*_2_ (g mg^–1^ min^–1^) is a pseudo-second-order rate constant of sorption, *K*_i_ (mg g^–1^ min^–0.5^) is the intraparticle diffusion rate, *X* (mg g^–1^) is the boundary layer diffusion effects (external film resistance), α (mg g^–1^ min^–1^) is the initial sorption rate, and β (g mg^–1^) is the desorption constant. The *k*_1_, *q*_e_, and *R*^2^ values for the PFORE model were computed using linear plots of log(*q*_e_ – *q_t_*) vs *t*. While the values of *k*_2_, *q*_e_, and *R*^2^ of the PSORE model were found using linear graph plots of *t*/*q*_*t*_ vs *t*.

The estimated findings for the PFORE and PSORE models are summarized in Table 3. The *R*^2^ values of PFORE and PSORE that have been given in Table 3 are in close proximity to one another. Furthermore, when compared to the PFORE model, the sorption process of CV obeys the PSORE model in terms of a higher *R*^2^ value. Besides, the consistency between the experimental (*q*_exp_) and the calculated (*q*_cal_) sorption capacities issued from the PSORE model and the experimental *q* (*q*_exp_) values supports this result. This result is consistent with the kinetic model fitting results of various pollutant-adsorbent systems.^74,75^ Overall, the chemisorption pathway regulates CV dye sorption onto the MPBC sorbent, including the valence force of sharing or exchanging electrons between sorbate molecules and sorbent.^76,77^

**Table 3:** Kinetics Modeling Parameters of CV Dye Sorption onto MPBC Sorbent

Kinetic models		Results
PFORE	*k*_1_ (min^–1^)	0.04
	*q*_e_ (mg g^–1^)	7.49
	*R*^2^	0.98
PSORE	*k*_2_ (g mg^–1^ min)	0.01
	*q*_e_ (mg g^–1^)	11.66
	*R*^2^	0.99
IPDE (Step I from 1 to 10 min)	*k*_i_ (mg g^–1^ min^0.5^)	2.72
	*X* (mg g^–1^)	–1.66
	*R*^2^	0.95
IPDE (Step I from 10 to 60 min)	*k*_i_ (mg g^–1^ min^0.5^)	0.81
	*X* (mg g^–1^)	4.58
	*R*^2^	0.96
IPDE Step III from (60 to 180 min)	*k*_i_ (mg g^–1^ min^0.5^)	0.03
	*X* (mg g^–1^)	10.60
	*R*^2^	0.85
Elovich equation	α (mg g^–1^ min^–1^)	4.62
	β (g mg^–1^)	0.50
	*R*^2^	0.95

Typically, the sorption process is a stepwise process, consisting of (i) external diffusion, (ii) intraparticle diffusion, (iii) and a sorption reaction. The intraparticle diffusion model (IPD) was studied by plotting *q*_*t*_ vs *t*^0.5^. If the plot passes via the origin (*C* = 0), IPD is the only rate-controlling step. Because the linear curve did not pass through the origin, pore diffusion is not just a rate-determining step. As the sorption process of CV presents a multilinear graph, the film diffusion and surface sorption may influence the CV sorption process onto the MPBC sorbent. With increasing dye concentration, the boundary layer width widened. The mass transfer rate to the external surface and the boundary layer width influenced the CV sorption.^78^

The Elovich model is the last explored kinetics model, matched with heterogeneous sorbent surface. Moreover, it is compatible with the chemisorption process.^79^ As stated in Table S1, the findings of high initial sorption rate and low desorption constant values strongly affirm the suitability of the Elovich model for the sorption of CV onto the MPBC sorbent.

#### Influence of Temperature (Thermodynamics Assay)

The influence of the environmental temperature aspect on the sorption profile of the CV dye onto the MPBC sorbent was carried out at various temperatures to understand the nature of the sorption process. The equilibrium sorption capacity of MPBC increased significantly from 12.1 mg g^–1^ (RE % = 90.8%) to 12.6 mg g^–1^ (RE % = 95.05%) as the temperature increased from 298.0 to 328.0 K, indicating the highest affinity of the MPBC sorbent for the CV dye at elevated temperatures. This intuitively confirms the endothermicity of CV sorption applying the MPBC sorbent.^80^ Certainly, the high tendency of CV molecules toward the MPBC sorbent as the temperature increases can be explained by a decrease in the thickness of the exterior boundary layer surrounding the MPBC sorbent and an increase in the activity of binding centers on the surface of the sorbent. This likely facilitated the movement of CV molecules, their diffusion within the sorbent’s pores, and their subsequent interaction with the available free binding centers.^81^

Importantly, the thermodynamics studies are capable of elaborating the sorption mechanism of CV molecules onto the MPBC sorbent (Figure 7c). The essential thermodynamics parameters of Δ*S*°, Δ*H*°, and Δ*G*° are calculated using eqs 12–15:
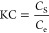
12
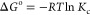
13
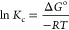
14
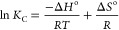
15where *K*_c_ is the equilibrium constant, *C*_s_ and *C*_e_ are equilibrium concentrations of the CV dye onto the sorbent surface and in an aqueous solution, respectively, Δ*G*° is the change in Gibbs free energy, change in entropy (Δ*S*°), and change in enthalpy (Δ*H*°). The thermodynamics functions for CV sorption on the MPBC sorbent were determined by plotting ln *K*_c_ against 1/*T*, and the results are displayed in Table S1. Clearly, the values of Δ*G*° decreased with increasing the solution temperature from 298.0 to 328.0 K, implying the spontaneous nature of the CV sorption onto the MPBC sorbent at elevated temperatures.^82^ The positive value of Δ*H*° presented that the sorption of CV onto MPBC sorbent was endothermic.^83^ Actually, the magnitude of Δ*H*° is valuable to denote a clear vision of the interaction mechanism between sorbent and sorbate in the solid-liquid interface system. Many studies have declared that the Δ*H*° value between 80.0 and 200.0 kJ mol^–1^ indicates chemisorption, whereas values below this range indicate physicosorption.^84^ From Table S1, the Δ*H*° value was 17.40 kJ mol^–1^, which is in accordance with the physical sorption domain. Moreover, the positive sign of Δ*S*° of 0.077 kJ mol^–1^ K^–1^ revealed an augmentation in the randomness of the sorption system as CV sorption onto the MPBC sorbent approached equilibrium.^85^

#### Influence of Competitors Ions

The influence of ionic strength on the sorption process is regarded as of relevant importance, considering the inevitable presence of other competitors (i.e., molecules and/or ions) in the industrial effluents.^86^ The highest RE % of 89.3% of the MPBC sorbent toward the CV dye was achieved in the lowest NaCl concentration (e.g., 5.0 g L^–1^), and it generally decreased (downward trend) as the concentration of NaCl increased until it reached its marginal value of 67.9% at a NaCl concentration of 45.0 g L^–1^, as summarized in Figure S2. Distinctly, a slight restriction in the sorption capacity of the MPBC sorbent from 11.91 mg g^–1^ (RE % = 89.3%) to 9.24 mg g^–1^ (RE % = 67.9%) toward the CV dye was observed in the presence of too high of a salt concentration. This inhibition phenomenon may be illustrated by different mechanisms as follows: (i) competition between the background electrolytes (i.e., Na^+^, and Cl^–^) and CV dye molecules, decreasing the electrostatic potential of MPBC sorbent surface, (ii) compressing the double electric layer and consequently electrostatic repelling of the CV dye from the MPBC surface, and (iii) the major influence of ionic strength on the activity coefficient of CV dye in the solution.^87,88^ Nonetheless, these results unexpectedly validated the MPBC sorbent’s resistance to ionic interference and demonstrated its suitability for further wastewater treatment applications.

#### Turnover Reusability Study

As the sorption process progresses, the sorption capacity of a used sorbent gradually decreases until it is ultimately depleted. Consequently, the effective regeneration scenario of the saturated sorbent simultaneously determined its actual economic and environmental advantages for future practical applications.^89^ The desorption property of the MPBC sorbent was evaluated, and the findings are shown in Table S2. Intriguingly, it is evident that the RE % of MPBC sorbent decreased from 91.1 to 84.2% after 5 sorption-desorption cycles of sorbent reusability. The observed decrease in RE % after the fifth sorption cycle may be attributed to the loss of the sorbent material and distortion (breakdown) in the MPBC sorbent network during the multiple sorption-desorption cycles, as well as the obstruction of occupied MPBC sorbent surface functional moieties by the species of unreleased CV dye molecules.^90^ Nonetheless, it was observed that the recyclability (regeneration efficiency) of 92.4% of the MPBC sorbent after the fifth cycle was significantly higher than that of other sorbents.^91,92^ Given its high sorption capacity, rapid RE % rate, and admirable adsorbability, the regenerated MPBC sorbent can be proposed as a highly cost-effective and convenient material for industrial wastewater treatment applications.

#### Comparative Study of Sorption Capacity of MPBC with Other Sorbents from the Literature

Table 4 lists the capacities of various sorbents for the sorption of CV dye. According to Table 4, sorbent has a significantly higher sorption capacity than other sorbents. Greater removal efficiency may be attributed to the greater availability of sorptive centers on the sorbent’s surface and within its porous structure.

**Table 4:** Sorption Capacities Comparison with Other Numerous Sorbents from the Literature

Sorbent	Experimental conditions	Sorption capacities of CV (mg g^–1^)	References
Calcium-alginate beads	[solid]/[solution] = 0.4 g L^–1^, CV concentration = 5.0–500.0 mg L^–1^, contact time = 120.0 min, *T* = 293.0 K, pH = 6.0	29.0	(93)
Activated carbon/Fe_3_O_4_ magnetic nanocomposite	[solid]/[solution] = 1.25 g L^–1^, CV concentration = 10.0–80.0 mg L^–1^, contact time = 60.0 min, *T* = 298.0 K, pH = 9.0	35.3	(94)
Magnetite nanoparticle decorated reduced graphene oxide	[solid]/[solution] = 0.2 g L^–1^, CV concentration = 5.0–20.0 mg L^–1^, contact time: 220.0 min, *T* = 298.0 K, pH = 10.0	62.0	(95)
Charred rice husk	[solid]/[solution] = 1.0 g L^–1^, CV concentration = 50.0–1000.0 mg L^–1^, contact time = 60.0 min, *T* = 298.0 K, pH = 10.0	62.85	(61)
Xanthated rice husk	[solid]/[solution] = 1.0 g L^–1^, CV concentration = 50.0–1000.0 mg L^–1^, contact time = 70.0 min, *T* = 298.0 K, pH = 10.0	90.02	(61)
Magnetic biochar	[solid]/[solution] = 2.0 g L^–1^, CV concentration = 50.0 mg L^–1^, contact time = 240.0 min, *T* = 303.0 K, pH = 6.0	111.48	(96)
Granular biopolymer-silica pillared clay composites	[solid]/[solution] = 2.5 g L^–1^, CV concentration = 50.0 mg L^–1^, contact time = 1440.0 min, *T* = 318.0 K, pH = 3.0	208.9	(97)
Date palm petioles-biochar	[solid]/[solution] = 1.0 g L^–1^, CV concentration = 5.0–500.0 mg L^–1^, contact time: 1440.0 min, *T* = 303.0 K, pH = 7.0	209.0	(50)
Alginate-Whey composite beads	[solid]/[solution] = 0.4 g L^–1^, CV concentration = 50.0–500.0 mg L^–1^, contact time: 8640.0 min, *T* = 303.0 K, pH = 6.0	220.0	(93)
Magnetic plastic waste-biomass char	[solid]/[solution] = 1.5 g L^–1^, CV concentration = 10.0–1000.0 mg L^–1^, contact time: 60.0 min, *T* = 298.0 K, pH = 10.5	256.41	Present study

#### Application of MPBC Sorbent for Crystal Violet Dye Removal from Spiked Wastewater

To further verify the practicability of the MPBC sorbent as a color collecting material, its sorption performance toward the CV dye was evaluated against real aqueous dyeing matrices, considering the complexity of the system caused by multiple components (i.e., inorganic cations/anions, organic matters, natural minerals, and biological constituent). The wastewater specimen was treated with the developed MPBC sorbent. The MPBC sorbent successfully captured the CV dye from real effluents with a little loss in the RE % from 85.6% (e.g., 5.0 mg L^–1^ of CV concentration) to 71.1% (e.g., 20.0 mg L^–1^ of CV concentration). The results verified the outstanding feasibility of economical MPBC in polluted industrial water treatment.

## Conclusion

This study presents the preparation of a magnetic char composite using plastic bottle waste (polyethylene terephthalate) and biomass. Following the findings from the preparation process, a LCA was used to evaluate the environmental impact of the preparation of these composite materials. Lastly, the produced magnetic char composite material was used for CV dye removal in water treatment, where the magnetic properties of these magnetic char composites were utilized to enhance separation in the water treatment application. Regarding the LCA, for 1 functional unit (1 kg of pomace leaves as feedstock), abiotic depletion of fossil fuels and global warming potential were quantified as 7.17 MJ and 0.63 kg CO_2_ equiv for the entire process.

The CV dye removal findings showed that a basic pH boosts the CV sorption process. However, the as-employed MPBC sorbent exhibited a high RE % toward the CV dye even at a solution pH value of 2.3, which proposed that sorption of the CV dye onto the MPBC sorbent may be dominated by other impactful mechanisms of pore-filling, H-bonding, and π-π stacking. It was determined that 4.0 g L^–1^ of the sorbent was the optimal sorption for 20.0 mg L^–1^ of CV dye sorbate. Among the three sorption isotherm models, the Langmuir isotherm model was shown to be the best fit in experimental data of CV sorption onto the MPBC sorbent (*R*^2^ = 0.99). Overall, the chemisorption pathway regulates CV dye sorption onto the MPBC sorbent, including the valence force of sharing or exchanging electrons between sorbate molecules and sorbent. The equilibrium sorption capacity of MPBC increased significantly from 12.1 mg g^–1^ (RE % = 90.8%) to 12.6 mg g^–1^ (RE % = 95.05%) as the temperature increased from 298.0 to 328.0 K, indicating the highest affinity of the MPBC sorbent for the CV dye at elevated temperatures. In the actual wastewater, the adsorption performance of the adsorbent material toward the CV dye should be investigated. Future work will investigate the use of these magnetic char composite materials in real wastewater treatment, using effluent mixtures and on a large scale.

## Supporting Information Available

Influence of initial CV dye concentration on sorption capacity (mg g^–1^) and RE % of MPBC sorbent; influence of NaCl concentration (ionic strength) on CV dye sorption (C0: 20.0 mg L^–1^, *T* = 25 ± 1 °C, *t* = 180.0 min, and SS = 150.0 rpm); thermodynamics modeling parameters of CV dye sorption onto MPBC sorbent; desorption findings of sorbed CV dye from MPBC sorbent after 5 sorption-desorption cycles (PDF)
