# Supplementary material for: Facile Synthesis and Life Cycle Assessment of Highly Active Magnetic Sorbent Composite Derived from Mixed Plastic and Biomass Waste for Water Remediation
Source: ACS Sustain Chem Eng. 2022 Sep 7;10(37):12433–47. doi: 10.1021/acssuschemeng.2c04095 (PMC9490754; doi:10.1021/acssuschemeng.2c04095)
Supplement: Supplementary file 1 — sc2c04095_si_001.pdf [file sc2c04095_si_001.pdf]

Supporting Information

Facile synthesis and life cycle assessment of highly active magnetic sorbent composite derived from mixed plastic and biomass waste for water remediation

Ahmed I. Osman [a*♣], Ahmed M. Elgarahy [b,c♣], Neha Mehta [a], Ala'a H. Al-Muhtaseb [d], Ahmed S. Al- Fatesh[e], David W. Rooney [a]

[a] School of Chemistry and Chemical Engineering, Queen's University Belfast, Belfast BT9 5AG, Northern Ireland, UK.

[b] Environmental Science Department, Faculty of Science, Port Said University, Port Said, Egypt.

[c] Egyptian propylene and polypropylene company (EPPC), Port-Said, Egypt.

[d] Department of Petroleum and Chemical Engineering, College of Engineering, Sultan Qaboos University, Muscat, Oman.

[e] Chemical Engineering Department, College of Engineering, King Saud University, Riyadh, 11421, Saudi Arabia.

♣ Equal contributions

Corresponding Author: Ahmed Osman

Email: aosmanahmed01@qub.ac.uk

Address: School of Chemistry and Chemical Engineering, Queen's University Belfast, David Keir Building, Stranmillis Road, Belfast BT9 5AG, Northern Ireland, United Kingdom

Fax: +44 2890 97 4687

Tel.: +44 2890 97 4412

Supporting Information includes 6 Pages, 2 Figures and 2 Tables.

1. Characterization techniques

Powder X-ray diffraction (XRD) was carried out using a PANalytical X'Pert Pro X-ray diffractometer. This diffractometer was equipped with a CuK_α X-ray source with a wavelength of 1.5405 Å. The diffractograms were collected up to 2θ = 80°. The X-ray tube was set at 40 kV and 40 mA. The morphology of the char composites' surface was characterized by transmission electron microscopy (TEM) (JEOL 2100 with high tension of 200 kV and a point resolution of 0.24 nm). XPS was performed in a ThermoFisher Scientific Instruments (East Grinstead, UK) with a quartz monochromator Al Kα radiation of energy 1486.6 eV. For the construction and fitting of synthetic peaks of high-resolution spectra, mixed Gaussian-Lorentzian functions with a Shirley-type background subtraction were used.

XPS spectra were obtained using multiprobe X-ray photoemission spectroscopy (XPS) (Omicron Nanotechnology, Germany) with a monochromatic Al Kα radiation (hν = 1486.6 eV) working at 15 kV, 20 mA. High-resolution XPS spectra were deconvoluted to individual components using Casa XPS software (Casa Software Ltd). The intrinsic carbon C 1 s peak at 284.6 eV was used as calibration. In order to avoid charging effect, sample surface was flooded with an electron beam during measurement. Zeta Analyzer NICOMP 380/ZLS made in the USA was utilized to investigate surface charge analysis. Zeta potential was measured for the magnetic char composites at conditions of E-Field=5.00 V/cm; Cell V=2.00; Cell Current: I_{max}= 0.86 and average of = 0.86.

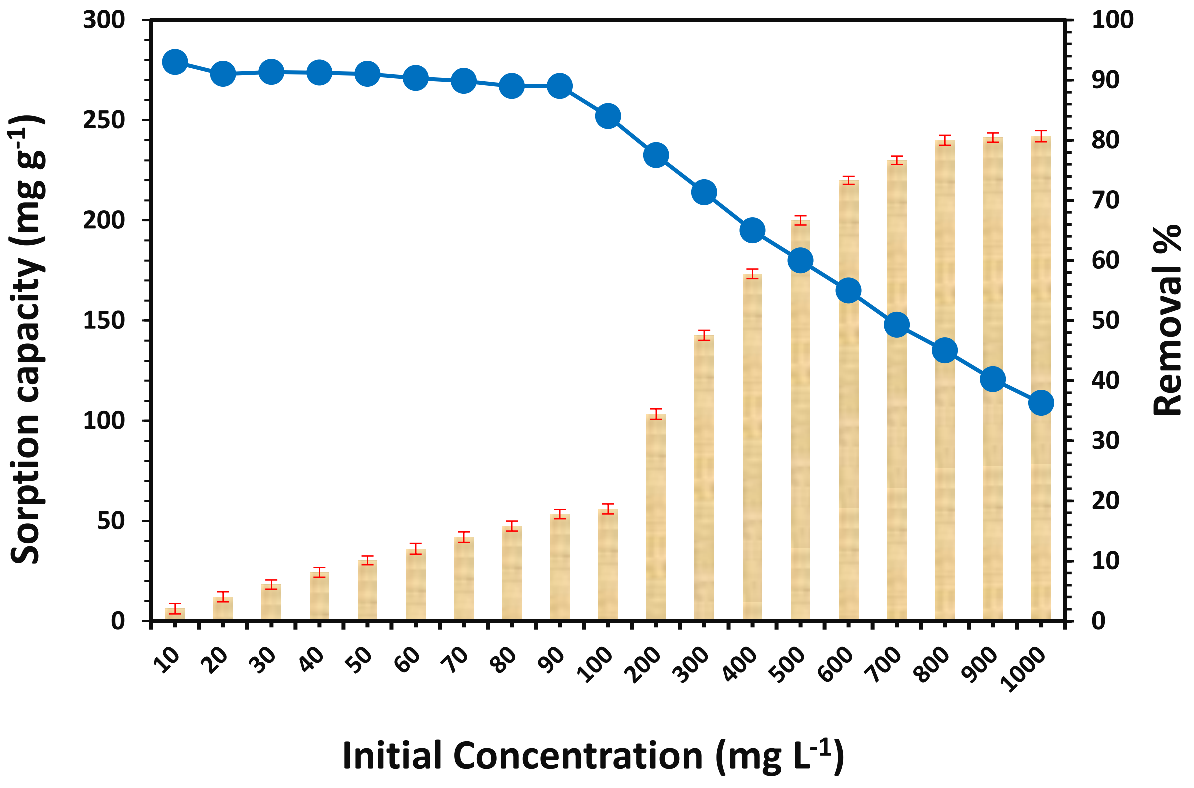

Figure S1: Influence of initial CV dye concentration on sorption capacity (mg g-1) and RE% of MPBC sorbent.

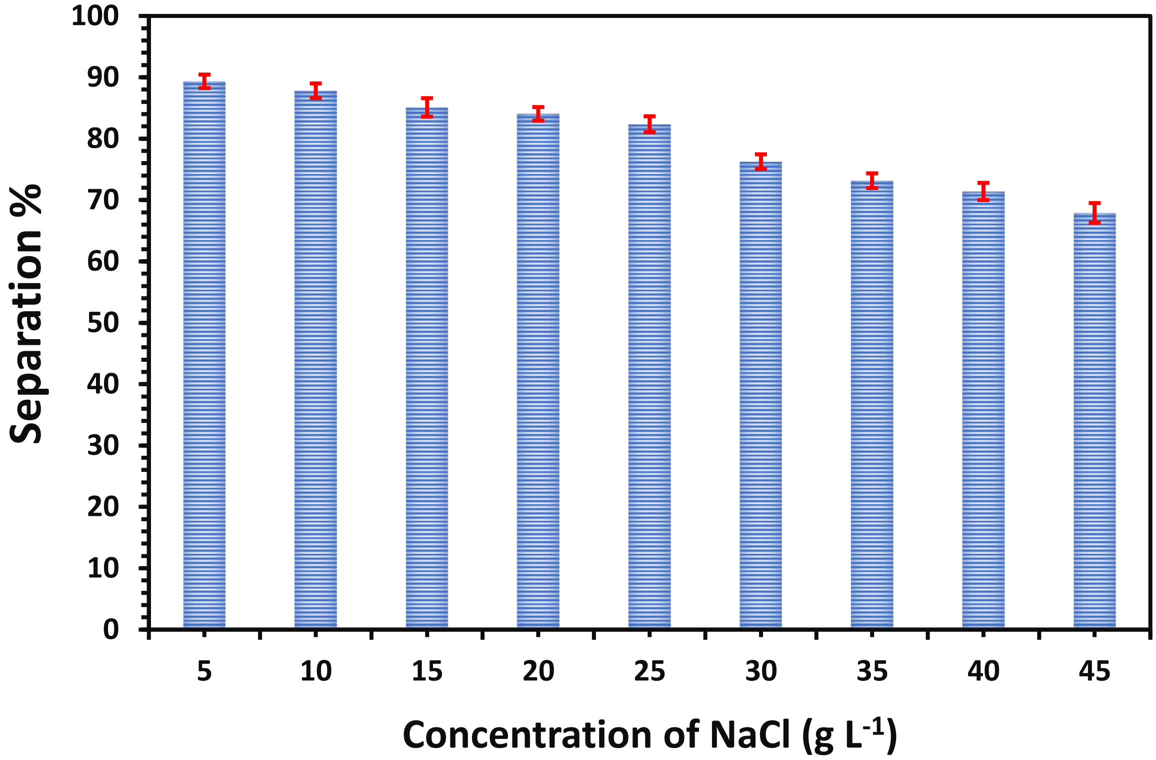

Figure S2: Influence of NaCl concentration (ionic strength) on CV dye sorption (C_0: 20.0 mg L^{-1}, T = 25 ± 1 °C, t = 180.0 min and SS = 150.0 rpm).

Table S1: Thermodynamics modeling parameters of CV dye sorption onto MPBC sorbent.

**Dye**	**ΔH° (kJ mol⁻¹)**	**ΔS° (kJ mol⁻¹ K⁻¹)**	**R^2**	**TΔS° (kJ mol⁻¹)**				**ΔG° (kJ mol⁻¹)**			
				298 K	308 K	318 K	328 K	298 K	308 K	318 K	328 K
**CV**	17.40	0.07	0.97	23.02	23.79	24.56	25.34	-5.62	-6.39	-7.16	-7.94

Table S2: Desorption findings of sorbed CV dye from MPBC sorbent after 5 times of sorption/desorption cycles.

**Sorption/desorption cycle**	**Crystal violet dye**		
	**Sorption capacity (mg g^{-1})**	**Removal (%)**	**DES (%)**
**First sorption operation**	12.1	91.1 %	-
**Cycle 1**	11.9	89.7 %	98.4 %
**Cycle 2**	11.6	87.6 %	96.1 %
**Cycle 3**	11.4	85.6 %	93.9 %
**Cycle 4**	11.1	83.8 %	91.9 %
